# Host genetic background influences diverse neurological responses to viral infection in mice

**DOI:** 10.1038/s41598-017-12477-2

**Published:** 2017-09-22

**Authors:** Candice L. Brinkmeyer-Langford, Raquel Rech, Katia Amstalden, Kelli J. Kochan, Andrew E. Hillhouse, Colin Young, C. Jane Welsh, David W. Threadgill

**Affiliations:** 1 Department of Veterinary Integrative Biosciences, Texas A&M University, College Station, Texas 77843 USA; 2 Department of Veterinary Pathobiology, Texas A&M University, College Station, Texas 77843 USA; 3 Texas A&M Institute for Genomic Sciences and Society, Texas A&M University, College Station, Texas 77843 USA; 4 Department of Molecular and Cellular Medicine, Texas A&M University, College Station, Texas 77843 USA

## Abstract

Infection by Theiler’s murine encephalomyelitis virus (TMEV) is a model for neurological outcomes caused by virus infection because it leads to diverse neurological conditions in mice, depending on the strain infected. To extend knowledge on the heterogeneous neurological outcomes caused by TMEV and identify new models of human neurological diseases associated with antecedent infections, we analyzed the phenotypic consequences of TMEV infection in the Collaborative Cross (CC) mouse population. We evaluated 5 different CC strains for outcomes of long-term infection (3 months) and acute vs. early chronic infection (7 vs. 28 days post-infection), using neurological and behavioral phenotyping tests and histology. We correlated phenotypic observations with haplotypes of genomic regions previously linked to TMEV susceptibility to test the hypothesis that genomic diversity within CC mice results in variable disease phenotypes in response to TMEV. None of the 5 strains analyzed had a response identical to that of any other CC strain or inbred strain for which prior data are available, indicating that strains of the CC can produce novel models of neurological disease. Thus, CC strains can be a powerful resource for studying how viral infection can cause different neurological outcomes depending on host genetic background.

## Introduction

Viral infections may precede the onset of several neurological conditions, including epilepsy, Parkinson’s disease (PD), multiple sclerosis (MS), and amyotrophic lateral sclerosis (ALS)^1–14^. For each of these diseases, a viral infection in childhood or young adulthood is thought to initiate a chain of immunological events that, along with other risk factors, result in a cumulative increased susceptibility to disease. The risk and clinical manifestations conferred by antecedent viral infection can vary greatly among individuals even when infected by the same virus. Experimental studies using mice have uncovered mechanisms of infection, but common inbred and transgenic strains of mice typically do not reflect the genetic diversity and phenotypic heterogeneity observed in human populations. Consequently, the mechanisms responsible for variable responses to viral infection in humans remain largely unknown.

Theiler’s murine encephalomyelitis virus (TMEV) is a naturally occurring neurotropic picornavirus affecting mice^15^. TMEV produces complications, which vary by mouse strain, that closely approximate human neurological conditions associated with antecedent viral infections. Moderately virulent strains (BeAn and DA) of TMEV establish a persistent infection resulting in demyelinating disease similar to MS in susceptible mouse strains such as SJL^16,17^ and, to a lesser extent, CBA^18^. In susceptible mouse strains, TMEV infection is biphasic. The early period of infection (first 4 weeks) is characterized as an acute inflammatory phase, eliciting a strong anti-TMEV antibody response^19^ along with an anti-TMEV T-cell response to clear the viral infection^20^. The later period of TMEV disease (from approximately 7 weeks post infection) is characterized by widespread neurodegeneration and chronic neuroinflammation^21,22^. In the TMEV-resistant strain C57BL/6, TMEV infection causes epileptic seizures, but these mice clear the virus from the central nervous system (CNS) and do not develop chronic demyelination as seen in SJL mice^23,24^. Depending on mouse strain, TMEV can infect motor neurons, resulting in lesions similar to ALS in humans^25^, or dopaminergic neurons in the substantia nigra of CBA mice, similar to PD^26,27^. Since infection-associated neurological conditions in mice vary depending on genetic background, TMEV infection can be used to model heterogeneous infection-associated human diseases to better understand the underlying genetic components.

Genetic and phenotypic variation underlying host response has been previously studied for influenza^28,29^, SARS-CoV^30^, Ebola^31^, and West Nile virus^32,33^ using the Collaborative Cross (CC) mouse genetic reference population. The CC was designed to model genetic heterogeneity of humans^34,35^, where each CC strain can be considered as a unique individual and the panel as a population of humans. In this study the utility of the CC was evaluated for its ability to reveal the spectrum of neurological phenotypes caused by infection by TMEV, based on genetic background. Our findings confirmed that the genetic diversity present in CC strains contributes to phenotypically diverse neurological conditions resulting from TMEV infection.

## Results

### TMEV RNA quantification

**Figure 1:**
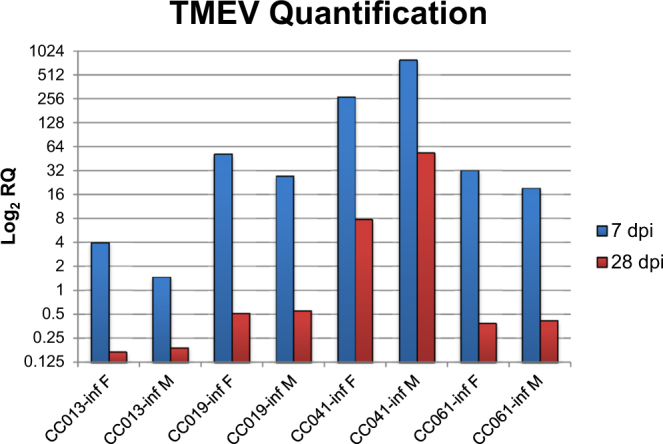
Brain TMEV levels were measured via qPCR in Group B mice at 7 and 28 dpi. Y-axis indicates log_2_ TMEV RQ values. Sample numbers for each group/sex were too low for statistical significance. For each strain, 2 females and 2 males, including 1 infected of each sex, were evaluated for 7 dpi and 2–3 mice of each sex per strain, including 1–2 infected per sex, were evaluated for 28 dpi.

A cohort of animals, hereafter referred to as Group B, was evaluated at 7 and 28 days post infection (dpi) to identify phenotypic differences related to the acute phase of TMEV infection. (Phenotypes of Group A mice were measured for ~90 days; see Materials and Methods.) We evaluated viral clearance in Group B mice by measuring TMEV transcript expression in the brain (Fig. 1). Infected females had higher expression than males for all strains except CC041. Overall, TMEV measurements at 7 dpi were highest in CC041 mice, while conversely, expression was lowest in CC013, followed by CC061 and CC019 mice, respectively. At 28 dpi, TMEV remained elevated in CC041 mice, while viral levels in all other strains had returned to background control levels.

### Weight change as indicator of response to infection

The 5 strains of mice varied in size even before infection, but initial (date of infection) weights did not predict their final weights or weight gain patterns. To allow direct comparisons of weight loss across strains, we calculated the changes in percent starting weight over the course of the experiment. CC013 and CC016 mice showed the greatest overall weight gains for each sex from day 0 to termination.

**Figure 2:**
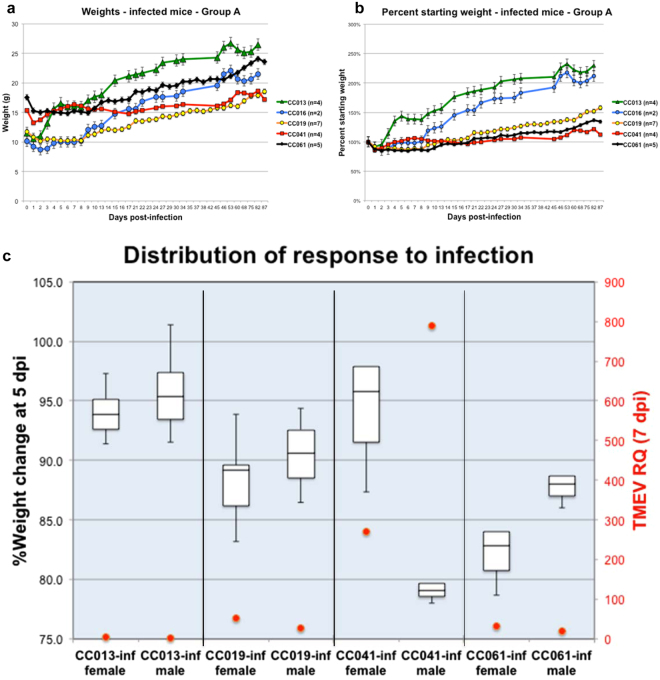
The varied responses to TMEV infection for mice from different CC strains and sexes are reflected by the percentage of weight lost/gained. (**a**) Shows weight changes in the different strains over the course of the experiment, while (**b**) shows percent weight changes based on the starting (day 0) weights of the mice. Group B percent weight change at 5 dpi, in relation to TMEV RQ levels, was used to evaluate differences in response to TMEV: in (**c**), the left y-axis displays the percent weight change at 5 dpi, relative to baseline (pre-infection); the right y-axis shows TMEV RQ levels at 7 dpi. Box plots show data quartiles and median; whiskers represent extreme data outcomes. Dots show corresponding levels of TMEV transcript (RQ values). A higher infection level (shown here as TMEV expression RQ) did not correlate with greater % weight change for CC041, which also showed gender-specific responses.

All infected mice lost weight after infection (Fig. 2). We evaluated weight loss at 5 dpi as a metric for comparing response to infection^29^. CC013 males and CC041 females had a weight loss of less than 10%, the lowest initial response to infection. CC041 males and CC061 females had weight loss of greater than 15%, the highest initial response to infection. CC013 male mice recovered most rapidly from the post-infection weight loss, surpassing their average pre-infection weights within 4 dpi on average. In contrast, female CC061 mice took longest to regain the weight (24 dpi, on average), while male CC061 mice regained the weight within an average of 13 dpi. The greatest difference in recovering weight gain between sexes was observed for CC041 strain. Sex-specific differences in response to TMEV infection have been previously described^36^ and it is likely that such differences influence post-infection weight.

### Survival

**Figure 3:**
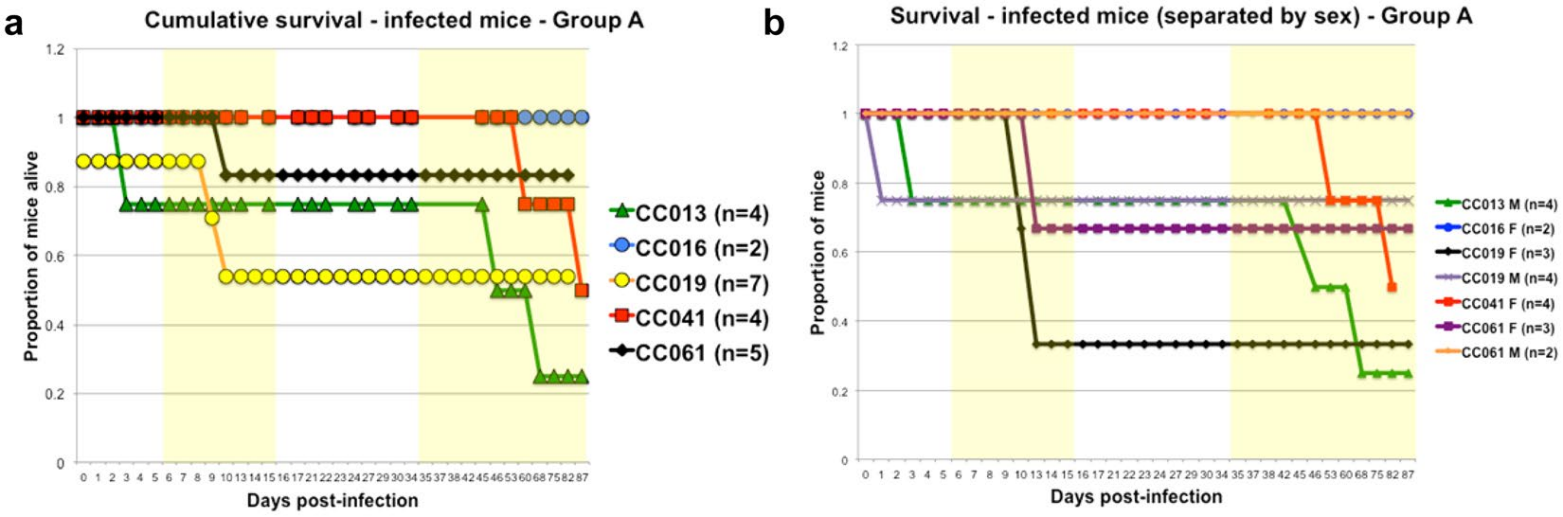
Survival rates of Group A mice varied by CC strain. (**a**) Shows a comparison of cumulative survival rates across all strains, while (**b**) further compares survival differences between sexes. The yellow-shaded boxes on the left of each panel highlight deaths occurring during the acute phase of TMEV disease, while those on the right highlights death that occurred during the chronic phase. The 0 dpi mortality for CC019 represents a mouse that died as a result of the infection procedure itself. One CC013 mouse died at 3 dpi for reasons that are unclear but likely related to infection.

Survival plots for Group A mice are shown in Fig. 3. The CC013 mice demonstrated signs of encephalitis throughout the experiment, and those that died were lost during both early and later stages of the infection. None of the CC016 mice died during the experiment. CC019 and CC061 mice that died during the experiment did so within the first two weeks. Thereafter, no other mortalities occurred for CC019 and CC061 mice. Mice of the CC041 strain fared well during the early infection period, but increased mortality was seen during the later, chronic stage of the infection.

All the mice that died were females. One CC019 male died immediately following infection, likely due to an adverse reaction to anesthesia rather than to the infection itself.

Of all mice in Group B, only one died: a CC019 female (infected) that died 13 dpi. However, this low mortality may be attributable to the small sample sizes.

### Seizures and clinical scoring

**Figure 4:**
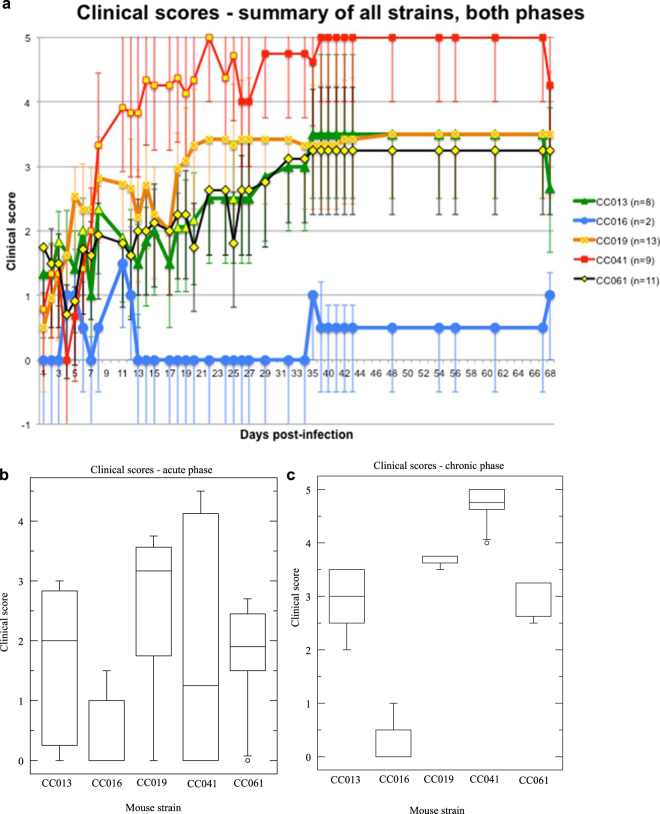
Clinical scores of TMEV-infected mice varied by CC strain. (**a**) Summarizes the clinical scores of all infected mice; data points with yellow centers are statistically significant (see Results). Clinical scores also varied by stage of TMEV-induced disease. Shown are ANOVA box plots constructed using Group A average daily scores for each strain during (**b**) the acute phase (6–15 dpi), and (**c**) the chronic phase (35 dpi and later).

TMEV infection resulted in strain-specific profiles of phenotypes, and the influence of strain on clinical score was statistically significant regardless of group (A vs. B), sex, or phase of disease. For clinical scoring purposes, the acute phase was defined as 6–15 dpi and the chronic phase as 35 dpi until the end of the experiment, 87–94 dpi^37^. Clinical scores were assigned as previously described, based on disease phase^38^: during the acute phase mice were scored based on ruffled appearance and hunched posture; during the chronic phase mice were scored based on hind limb weakness, gait and postural instability. Data are presented in Fig. 4 and Supplementary Table 4.

#### TMEV infection of CC013 mice results in seizure with limb impairment

All CC013 mice experienced seizures within 3–6 dpi, with Racine scores ranging from 3–5. These seizures generally lasted >1 minute. Seizure activity included forelimb clonus, drooling, and falling over. During the acute phase of infection, CC013 mice had piloerection that resolved by 28 dpi (but recurred around 35 dpi in Group A mice). At 7 dpi, all CC013 mice demonstrated hunched postures that resolved by 18 dpi (but returned at 35 dpi in Group A mice). Infected mice from this strain spent less time grooming themselves than controls (84%, or 90.8 seconds less, on average). CC013 mice demonstrated forelimb weakness during the inverted metal grate test: these mice consistently released the grate first with their front paws, holding on for a few more seconds with their hind paws.

Infection had a significant effect on the phenotype of CC013 mice during the early acute phase at 3, 4, 6, 8, and 11 dpi (*p* ≤ 0.05). The impact of infection was again significant on 17–20 and 25 dpi (*p* ≤ 0.05)but was not found to be significant thereafter. Sex played a significant role only on 3 dpi (*p* = 0.0161).

#### TMEV infection in CC016 results in seizures without limb impairment

Seizures in CC016 mice occurred within 3–6 dpi, with Racine scores ranging from 2–4. These seizures generally lasted <20 seconds. Piloerection was not noticeable in CC016, though these mice showed some slight hunching at 35 dpi. CC016 mice showed no signs of weakness or limb impairment.

#### TMEV infection in CC019 results in paralysis and increased lethality

In CC019 mice, subclinical seizure activities, including limb clonus and twisting behaviors, were observed during handling up to 10 dpi. CC019 infected mice displayed ruffling during the early acute phase of infection only, though the mice of this strain that died did so during this early period and were particularly ruffled 1–2 days prior to death. The most pronounced hunching was observed in infected male CC019 mice of Group A throughout the entire experiment period. Three of the 6 infected female CC019 mice also showed extreme hunching and died by 10 dpi, but the remaining 3 infected females did not demonstrate as pronounced a hunched back as the males. Infected mice of the CC019 strain spent slightly *more* time grooming themselves (an average of 11.5 seconds, or 22%, more) than did uninfected sex-matched controls of the same strain. Of infected CC019 mice, 22% displayed forelimb weakness in the first 2 weeks p.i. Also within the first 2 weeks p.i., 67% of infected CC019 females developed severe hind limb weakness or paralysis and died, and all infected CC019 males developed hind limb weakness or paralysis. All CC019 infected mice of Group A eventually stabilized, with no change in strength beyond a mild, brief “recovery of function” around 30 dpi.

Throughout the experiments, infection had a significant (or nearly significant) effect on the clinical scores of CC019 mice (*p* ≤ 0.05). Additionally, sex (*p* = 0.0247) was a significant contributor to clinical score at 2 dpi.

#### TMEV infection in CC041 results in debilitating limb paralysis

Seizures and piloerection were not observed in CC041 mice. These mice occasionally displayed hunched postures throughout the disease course starting from 7 dpi, but the hunching was not observed every day and probably was related to compensation for hind limb weakness. Infected mice from this strain spent less time grooming themselves than controls did (70.2 seconds, or 80% less, on average). CC041 mice demonstrated the most profound, debilitating hind limb weakness of the 5 strains studied. Evidence of deficiency in limb strength was observed from 8 dpi for all infected CC041 mice. By 11 dpi, 50% of infected CC041 mice were paralyzed in one or both hind legs, and by 20 dpi all dragged one or both hind legs.

Infection had a significant effect on the clinical score of CC041 mice throughout the acute phase and beyond, from 1 dpi through 25 dpi except for 4 and 5 dpi (*p* ≤ 0.05). Sex had a significant effect at 1 dpi (*p* = 0.0077).

#### TMEV infection in CC061 results in increased signs of encephalitis

Infected CC061 mice demonstrated forelimb clonus and grasping at their hind legs when lifted. These mice displayed ruffling during the early acute phase of infection only, and CC061 mice that died did so during this early period and were especially ruffled 1–2 days prior to death. Infected CC061 mice exhibited hunching up to 21 dpi and spent less time grooming themselves than uninfected controls did (48.0 seconds, or 29% less, on average). However, these mice did not have paralysis.

For CC061 mice, infection was a significant contributor to clinical scores throughout the disease throughout the experiment (*p* ≤ 0.05). Sex was also significant at 2 and 3 dpi (*p* = 0.0149).

### Gait assessment

#### Foot print

We compared stride measurements from infected and uninfected mice of the same strain in Group A, normalized to measurements on day 1. Overall width of hind limb stride was lower for infected CC013, CC019, and CC041 mice, particularly during weeks 10–13 p.i. CC061 stride widths were the least affected by infection. Hind limb stride lengths were lower in infected CC013, CC041, and CC061 mice, while CC019 mice showed little change in stride lengths regardless of infection status. For all foot print measurements, hind limb stride lengths decreased much more for infected CC041 mice than for infected mice in the other strains, reflecting the hind limb paralysis seen for this strain after TMEV infection.

**Figure 5:**
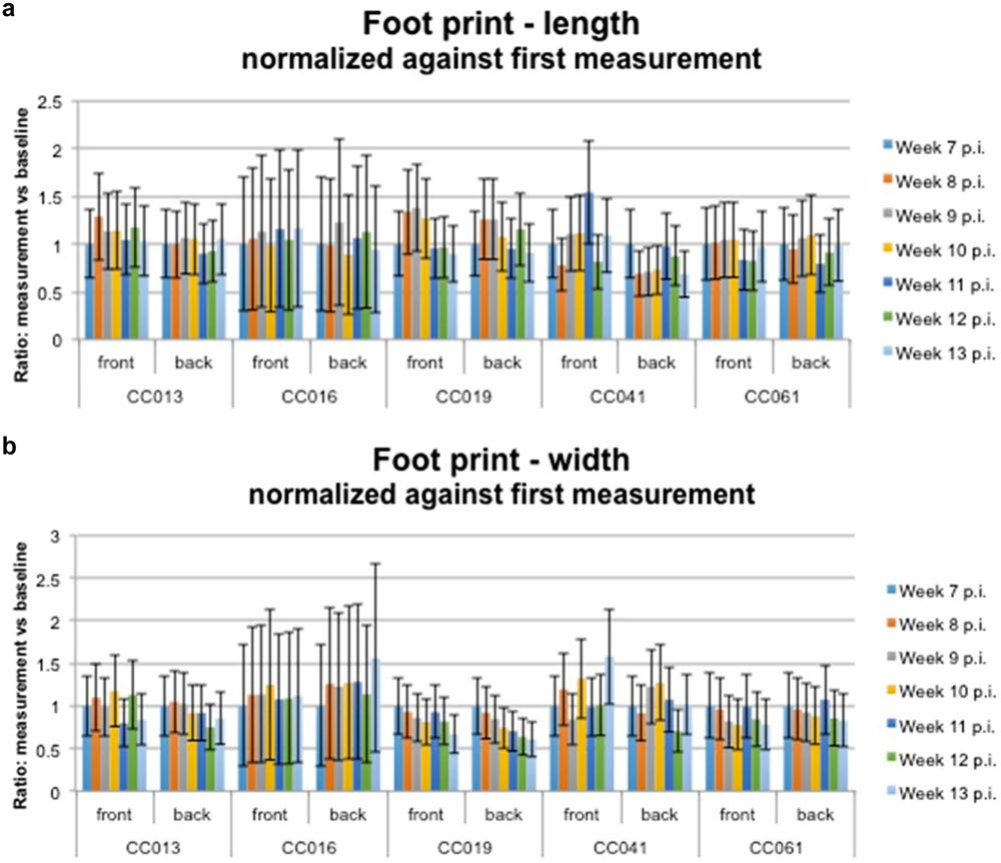
Foot print data showing variation in hind limb stride lengths and widths for infected mice. (**a** and **b**) Show variation in stride lengths and widths, respectively, based on measurements normalized to the first measurement (Week 7 p.i.). Error bars show standard error (S.E.M.), calculated as standard deviation divided by the square root of *n* (number of animals).

Overall, the genetic background of CC strains significantly influenced both fore and hind limb stride lengths and widths. Sex and infection also significantly affected stride length and width variation across strains. These differences likely reflect the strain-specific responses to TMEV infection: the spinal cord and hind limbs were more strongly affected in some mice (e.g. CC041), while the forelimb regions and brain were more affected in other mice (e.g. CC013). Furthermore, sex-specific differences in immune response were more prominent in some strains, e.g. CC041. Foot print data are summarized in Fig. 5. Foot print data statistics are presented in Supplementary Table 1.

#### DigiGait

DigiGait enables the objective, precise measurement of 32 gait parameters to provide a comprehensive evaluation of gait and related aspects such as ataxia and coordination. All measures of gait kinetics obtained via DigiGait varied markedly among CC lines. Sex-specific phenotypes also were observed, especially for CC019 and CC041. (See Supplementary Table 2 for *p*-values and other measurement data; DigiGait parameters are italicized in the text below).

CC013: The gaits of infected and uninfected CC013 mice did not appear to be different when viewed with the naked eye, but variations in gait parameters were measurable via DigiGait, showing that subtle differences can exist between different phenotypic responses to infection. Sex significantly influenced *brake* time, *stride length coefficient of variation* (CV; measured as percentage), and *ataxia* measurements for the forelimbs of CC013 mice. Infection also significantly influenced *brake* time measurements. In the hind limbs, *percent swing stride* and *percent stance stride*, reflecting the amount of time the paws were in the air or in contact with the treadmill, respectively, were significantly altered by infection. *Stride frequency* was significantly influenced by sex: females took more steps per second than males.

CC019: In CC019 mice, TMEV infection increased *paw angle variability*, decreased *overlap distance*, and increased *axis distance*. In hind limbs, it significantly decreased *brake* time, *percent brake stride*, and *percent brake stance*, and it increased *propel*, *percent propel stride*, and *percent propel stance*. Infection also increased *paw angle variability*, *stride length variability*, and *number of steps*, and it reduced *overlap* and *midline distances*. *Ataxia* coefficient decreased in infected females but increased in infected males. Taken together, these findings indicate that TMEV infection resulted in gait patterns reflecting postural instability and variable acceleration/deceleration, possibly indicating deficits in neuromuscular control and coordination.

Sex differences in phenotypes were especially pronounced in the CC019 strain, both in controls and in infected mice. Sex differences in response to TMEV infection were particularly obvious, and in fact female and male gait parameters were often affected in opposite directions. Infected female mice had increased measurements for *swing, swing stride*, and *stride*, while the measurements for these same parameters decreased in infected male mice. Moreover, infected females had decreased values for *stance/swing, step angle CV*, and *axis distance*, whereas infected males showed increases for these measurements. In both sexes, infection increased *paw angle variability* and decreased *overlap distance*; the changes were more dramatic for males than females. Significant sex-by-infection interactions showed that, for the hind limbs, infected females had more substantial changes in propulsion (shown by large increases in *propel, percent propel stride*, and *percent propel stance*) and deceleration (decreases in *brake time, percent brake stride*, and *percent brake stance*) than their male counterparts. Infected males exhibited more significant increases in *paw angle* and *stride length variability* than infected females, along with significant decreases in *overlap distance* and *ataxia* measurements.

CC041: As with CC019 mice, sex often influenced gait parameters in opposite directions in CC041 females and males. Infection significantly reduced the duration of the swing phase (*swing, percent swing stride*) in the forelimbs of females. The opposite was true for males. The opposite of swing time is stance time, and *percent stance stride* in the forelimbs increased for infected females but decreased for infected males. Similarly, *stride* times for the hind limbs – the sum of stance and swing durations – were significantly affected by sex; female stride times increased but male times decreased with infection. *Brake* time and *percent brake stride* were significantly affected by infection for both sexes, increasing in the forelimbs and decreasing in the hind limbs. Furthermore, *percent brake stance* in the forelimbs was influenced by both infection and sex; infection increased values in both sexes, and this increase was substantially greater for male mice. *Percent propel stride* and *percent propel stance* in the forelimbs decreased for infected mice of both sexes, more dramatically for males than females, indicating that infected mice (especially males) spent less time propelling themselves forward (versus slowing down) while walking. Rates of change for propulsion and deceleration are reflected in the DigiGait parameters *max* and *min dA/dt*; both of these parameters were significantly influenced by sex and infection in the hind limbs of CC041 mice.

Infection and sex also affected measures of neuromuscular control in CC041 mice, observed in terms of gait coordination. Measurements of *stance/swing* in the fore and hind limbs were significantly influenced by sex; for the forelimbs, infection increased values for infected females but decreased them for infected males; for the hind limbs, values decreased for infected females and males. This measurement, which reflects the ratio of swing to stance phase time, should be the same for fore and hind limbs. The differences for different limbs indicate gait asymmetry. Indeed, the DigiGait parameter *gait symmetry* was very significantly affected by both infection and sex across all limbs, increasing with infection in both sexes (though not enough for obvious pathological implications). Other measurements of gait consistency and symmetry, *paw area* and *paw area variability at peak stance*, further indicated gait asymmetry in this strain. *Paw area at peak stance* was significantly affected by sex and infection in both fore and hind limbs, and *paw area variability* was significantly affected by sex in the hind limbs. *Paw area* decreased with infection in the forelimbs but increased with infection in the hind limbs of females; *paw area* decreased in the forelimbs but was unaffected in the hind limbs of infected males. *Paw area variability* increased for the hind limbs of infected females but decreased for those of infected males. Paw *overlap distance*, defined as the overlap extent of ipsilateral fore and hind paws, decreased significantly for both sexes, especially females. *Paw placement positioning*, defined as the extent of overlap of fore and hind paw “prints,” increased for the forelimbs of infected males. *Midline* and *axis distances* were also significantly affected by sex. Hind limb *midline distances* decreased for infected females but increased for infected males, indicating different amounts of “reach” for the rear paws of infected mice.

Differences in body sizes of female and male mice were considered when evaluating DigiGait results. Not surprisingly, *stride length* differed by sex, though only in the hind limbs, where it increased for infected females and decreased for infected males. *Stride frequency* (steps per second), however, was significantly affected by sex for both fore and hind limbs, and significantly affected by infection for the forelimbs only. *Stride frequency* increased for the forelimbs but decreased for the hind limbs of infected females; it increased for both fore and hind limbs of infected males. These *stride frequency* observations are unlikely to have been caused by differences in mouse body size, as *stride frequency* has more to do with cadence than step or limb sizes, especially when walking on a treadmill as with DigiGait.

CC061: Both infected and uninfected CC061 mice appeared to have similar gaits, but subtle differences could be appreciated by evaluating individual gait parameters via DigiGait. For the forelimbs, the gait parameters *percent brake stride, percent brake stance, percent propel stance*, and *paw angle variability* were all significantly affected by TMEV infection, while *stride length variation* and *stride length CV* were significantly affected by sex, both decreasing in infected males. The hind limb parameters *paw area variability* and *gait symmetry* were significantly affected by sex, with the former increasing and the latter decreasing in infected males; *gait symmetry* was also significantly affected by infection.

### Rotarod

**Figure 6:**
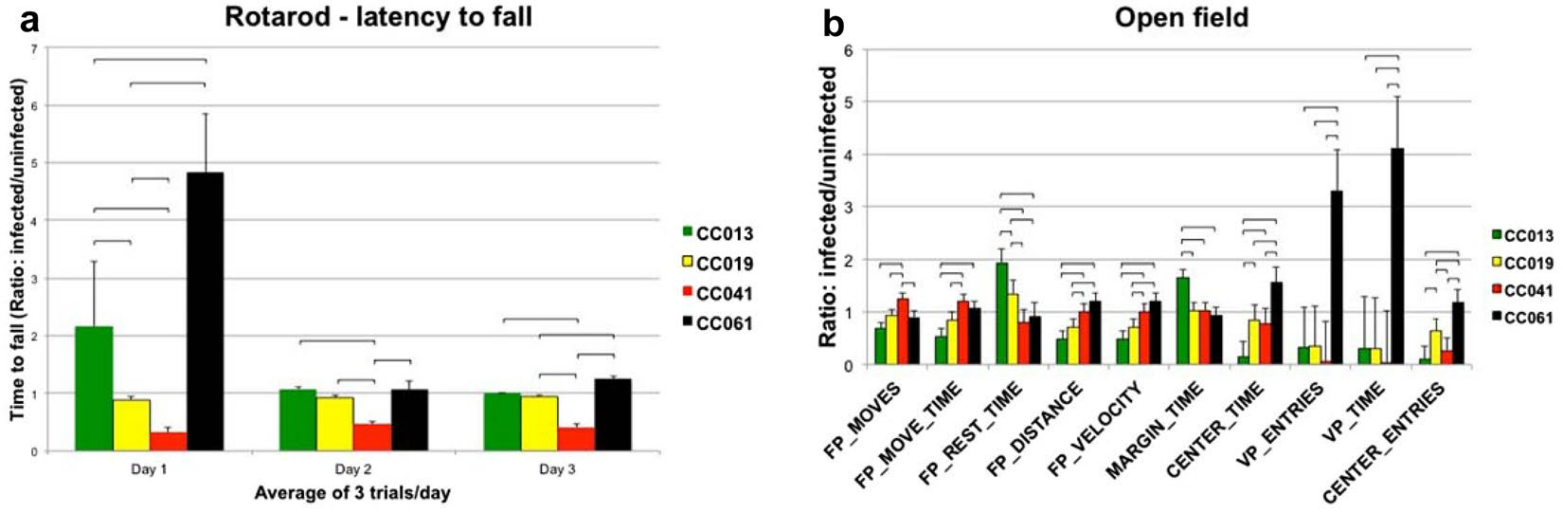
Rotarod and open field behavioral comparisons across CC strains of Group A. CC016 mice were not included because there were no uninfected CC016 mice for comparison. (**a**) Rotarod measurements: the y-axis indicates the ratio between latency-to-fall measurements of infected vs. uninfected mice, using the average measurements from 3 trials per day over 3 days. (**b**) Open field results displayed as ratio of observed values for infected mice vs. uninfected mice. Fisher’s post hoc test, LSD between strains showed significant (*p* < 0.05) differences between strains, which are indicated here as brackets encompassing the two strains involved. Numbers of mice included in these experiments: CC013 – 5, CC019 – 6, CC041 – 8, CC061 – 5. FP = floor plane; VP = vertical plane. Error bars show S.E.M.

The rotarod assessment detects motor dysfunction in mice during the chronic period of TMEV infection^38,39^. Rotarod findings are summarized in Fig. 6. Rotarod tests were performed to provide some consistency with previous TMEV studies, in which rotarod has featured prominently for evaluating phenotype; however, we did not find rotarod as useful as updated methods (e.g. DigiGait). Most uninfected mice remained on the rod without falling for 300 seconds. By the third day of testing, infected CC013 and CC016 mice performed as well on the rotarod assessment as uninfected controls, and infected CC019 and CC061 mice spent >280 seconds on the rotarod before falling. Compared with infected CC061 mice, uninfected CC061 mice spent *less* time on the rotarod before falling (an average of 57 seconds less per trial on day 3, 60–70% as long as uninfected mice from other strains). Infected CC041 mice showed an average latency to fall of 117 seconds (range: 42–170 seconds), a nearly 60% decrease from uninfected CC041 mice.

No significant sex differences were observed for any strain.

Based on our other observations, the reduced performance of the infected CC041 mice is likely attributable to their response to infection, while the performance of the uninfected CC061 mice may be linked to differences in behavioral responses to anxiety as exhibited in open field tests, described below.

### Open field

Results of open field tests have been previously shown to be influenced by the genetic backgrounds of inbred mice^40^; they are also influenced by TMEV infection^41^. In the present study, open field analyses revealed varied effects of TMEV infection on the anxiety and exploratory behaviors of mice of the different CC strains. All open field results are summarized in Fig. 6 and Supplementary Table 3.

Average time spent moving on the floor plane (horizontally) decreased by nearly half in infected CC013 mice, but it increased slightly for infected CC041 mice. TMEV infection had little effect on floor plane movement in CC019 and CC061 mice. Compared to uninfected controls, the average horizontal distances traveled by the mice, as well as the velocity with which they traveled, decreased for infected CC013 and CC019 mice but remained the same for CC041 and CC061 mice. Not surprisingly, infected CC013 and CC019 mice spent more time resting than their uninfected counterparts; however, in the CC041 and CC061 strains, rest times differed little between infected and uninfected mice.

Time spent on the margins of the open field arena can show how TMEV infection affects anxiety behaviors^41^. In general, margin time reflects anxiety and a reduced desire to explore^42^. On average, uninfected CC013 mice stayed nearly 7 times as long in the center of the arena than did infected mice of the same strain. For CC013 and CC041 strains, more uninfected than infected mice ventured into the center for any length of time at all – on average, nearly 9 times more often for CC013 mice, and 3.7 times more for CC041 mice. However, infected mice of the other two strains did not spend substantially more time on the margins. In fact, infected CC019 and CC061 mice spent significantly more time in the center of the arena. Infected CC061 mice also *entered* the center of the arena more than uninfected mice, unlike infected mice of the other strains.

Vertical plane entries (“rearing”) reflect exploratory behavior, which can also be affected by sickness. Infected CC013, CC019, and CC041 strains showed reduced vertical plane entries; this effect was much more pronounced in CC041 mice than in the other 2 strains. Interestingly, infected CC061 mice spent significantly more time, and had more entries, in the vertical plane than uninfected CC061 mice.

### Histological evaluation of central nervous system

Histological evaluation of female and male infected mice of all strains revealed morphologic changes not observed in any control mice. Importantly, the histological profiles of the CC strains were distinct from each other and from those described in previous TMEV studies involving inbred strains (SJL/J, CBA, and C57BL/6)^38,43–45^.

#### CC013

**Figure 7:**
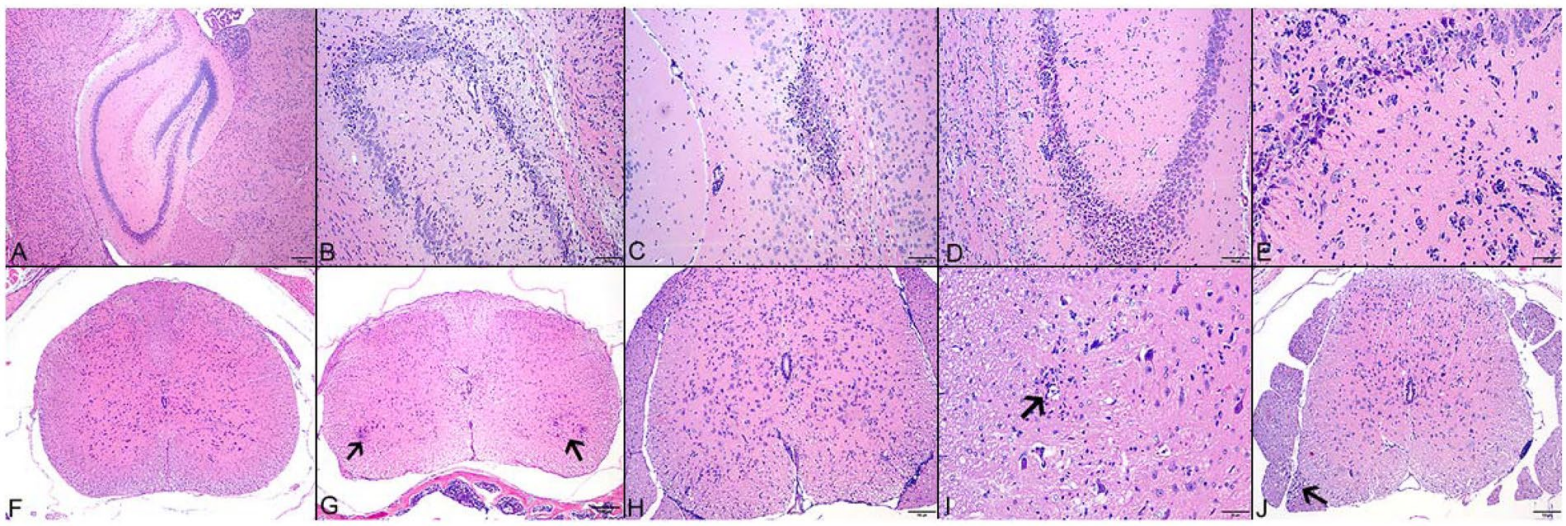
(**A**) Hippocampus, control (CC013). (**B**) Hippocampus, CC013 (7 dpi): Lymphocytic encephalitis with neuronal necrosis. (**C**) Subcortical necrosis with mineralization, CC019 (28 dpi). (**D**) (CC041) and (**E**) (CC061): Hippocampus (7 dpi); lymphocytic encephalitis with neuronal necrosis. (**F**) Spinal cord, control (CC019). (**G)** Spinal cord, CC013 (7 dpi): Bilateral ventral lymphocytic poliomyelitis (shown with arrows). (**H**) Spinal cord, CC019 (7 dpi): Bilateral ventral lymphocytic meningomyelitis. (**I**) Spinal cord, CC041 (28 dpi): Lymphocytic myelitis with neuronophagia (shown with arrow). (**J**) Spinal cord, CC061 (28 dpi): Lymphocytic meningomyelitis with poliradiculoneuropathy (shown with arrow). H&E stain.

At 7 dpi and 28 dpi, the lesions consisted of lymphocytic encephalitis in the subcortical regions of levels 2, 3 and 4 with focal to focally extensive areas of parenchymal necrosis, mainly in the hippocampus (CA1 region) and around lateral ventricles (Fig. 7B). Areas of necrosis were often associated with foci of mineralization. Level 6 was unremarkable in all infected mice. The lesions in the spinal cord were subtler than those in the brain. They were present only at 7 dpi and consisted of mild meningopoliomyelitis and radiculoneuritis.

#### CC016

No histologic lesions were observed in this strain.

#### CC019

Although lesions in the brain also consisted of lymphocytic polioencephalitis, hippocampal necrosis and mineralization, predominantly at levels 3 and 4, these lesions were milder than those in CC013 mice. In the spinal cord, lesions were predominantly inflammatory at 7 dpi, and they consisted of lymphocytic meningomyelitis centered in the ventral horns and axonal degeneration in the ventral funiculus. By 28 dpi, lesions were consistently degenerative in the white matter, characterized by axonal degeneration in the ventral funiculi and nerve roots. Both infected (4/5) and control mice (4/4) had mild hydrocephalus, which has been previously observed for this strain (http://csbio.unc.edu/CCstatus/index.py?run=availableLines) and was considered a background lesion^46^.

#### CC041

Overall, lesions were more severe in CC041 mice than in the strains CC013 and CC019. At 7 dpi, this strain developed subcortical lymphocytic encephalitis with necrosis of the neuropil around the lateral ventricles and hippocampus, mostly at levels 3 and 4, but also affecting levels 2 and 6. At 28 dpi, lesions consisted of mineralization of the hippocampus. In the spinal cord, the most predominant lesions at 7 dpi included lymphocytic meningomyelitis with neuronal necrosis, mostly in the ventral horns (Fig. 7I). In contrast, at 28 dpi lesions were characterized by neuronal necrosis and loss of the ventral horns, axonal degeneration of the nerve roots and secondary group myofiber atrophy of the adjacent musculature, mostly in the lumbar segments.

#### CC061

Mild lymphocytic meningoencephalitis was present in most levels at 7 dpi, but predominantly at levels 3 and 4; it was accompanied by hippocampal necrosis and mineralization to a lesser extent. Minimal to mild lymphocytic meningomyelitis was observed at 28 dpi, most prominent in the lumbar area of female mice, and was accompanied by mild polyradiculoneuropathy. Male mice did not present lesions in any segments of the spinal cord at 28 dpi.

## Discussion

In humans, viral infections can precede neurological conditions, sometimes by decades. However, not all people infected by a given virus will ultimately develop the associated neurological condition, and people can develop different neurological conditions from the same viral agent. For example, Guillain-Barré syndrome and multiple sclerosis have each been connected to antecedent infections by Epstein-Barr virus^47–49^. Guillain-Barré syndrome is also associated with infections, for example those by cytomegalovirus and Zika virus^50–53^. In this study, we evaluated five genetically distinct CC strains for their variability in response to TMEV, and we confirmed that individual strains of mice display different clinical characteristics indicative of diverse neurological pathologies.

### Phenotypic diversity

Each of the 5 CC strains responded uniquely to TMEV infection. None demonstrated TMEV-induced phenotypes exactly like those described for the inbred strains previously studied, demonstrating the utility of the CC for detecting and describing novel phenotypic profiles.

TMEV has historically been considered to affect the spinal cord more than the brain, but in the CC013 strain the brain was more affected, as demonstrated both phenotypically and histologically. The severe seizures experienced by CC013 mice during the acute phase of TMEV infection could explain the extensive necrosis found in the hippocampus, as TMEV-induced seizure activity has been linked to hippocampal damage and memory loss^54–57^. Also, these mice demonstrated certain behaviors, such as releasing the forelimbs first when held inverted on a grate, in keeping with the histological findings of neuronal death in the brain but not spinal cord. Furthermore, the relatively subtle gait changes in infected CC013 mice apparently resulted from injuries sustained in the CNS as a result of the epileptic seizures exhibited by this strain rather than lesions in the spinal cord, as no lesions were found in the spinal cords of CC013 mice at 28 dpi. The response of CC013 mice to TMEV was also distinct from those of previously studied inbred strains: 3 CC013 mice (out of 18 total) died during one phase or the other of TMEV infection, and based on the ongoing signs of encephalitis, these mice experienced a persistent state of inflammation rather than the typical immune shifts seen in TMEV infection^58,59^.

Infected CC016 mice reacted similarly to C57BL/6 mice infected with TMEV^23,45^, exhibiting seizures in the first few days after infection followed by apparent viral clearance as evidenced by the lack of additional clinical signs and the fact that all CC016 mice survived. CC016 mice did not have any histologic lesions. Altogether these findings may indicate that the response of CC016 mice to TMEV infection was most similar to that of C57BL/6; however, more mice of this strain need to be evaluated before reaching a conclusion.

CC019 mice were largely able to recover from temporary paralysis experienced shortly after the acute phase of the disease, though stride measurements via foot print indicated a subtle yet progressive weakening of the hind limbs. These clinical signs correlate with inflammatory changes at 7 dpi and degenerative lesions in the spinal cord at 28 dpi in this strain. Furthermore, DigiGait measurements suggest that the reduced neuromuscular control experienced by TMEV-infected mice caused reduced coordination in males, while the gait process itself – e.g., propulsion and deceleration – was affected in infected females. These differences in gait suggest different mechanisms underlie ambulation changes in females and males; however, no histological differences were observed between sexes. Taken together, these observations indicate that CC019 mice experienced a disease course unlike that of typical TMEV-sensitive strains such as SJL, for which limb weakness is progressive and occurs later in the disease and the sexes are histologically quite different.

Of the CC strains evaluated in this study, the TMEV response of CC041 most closely resembled (but was not exactly like) that of SJL mice. All CC041 mice had inflammatory lesions at 7 dpi but did not present clinical signs and survived the acute phase of infection. The distinct weight-loss difference between CC041 females and males at 5 dpi suggests sex differences in the early responses to TMEV infection in this strain. Estrogen is a modulator of TMEV infection^60–62^, and typically estrogen *protects* against TMEV pathogenesis: SJL females clear the virus better than SJL males and therefore develop less inflammation^36^. In this regard, CC041 mice resemble SJL mice in their initial response to TMEV infection. Next in the CC041 mice, motor neuron damage manifested as dramatic hind limb paralysis within 10 dpi, without full restoration of function. This paralysis correlates with lesions at 28 dpi characterized by degenerative changes in the ventral gray and white matter spinal cord and secondary atrophy of the skeletal myofibers in the lumbar segments. In the CC041 strain, gait parameter measurements indicated that TMEV-infected male mice experienced greater loss of strength than infected females did. Sex-specific differences in *swing* parameter measurements may be attributed to pain and/or reduced mobility in the male mice^63,64^. Although SJL mice infected with TMEV also experience reduced mobility during the chronic phase, for SJLs the decline is more gradual, with gait changes becoming apparent closer to 49 dpi rather than within 10 dpi as in CC041.

Finally, CC061 mice also exhibited phenotypes unlike anything observed previously in relation to TMEV infection. Clinical signs were not observed in CC061 mice during the acute phase but the mice had mild encephalomyelitis. During the chronic phase these mice exhibited a persistent waddling gait. This may be linked to persistent lesions in this strain of mice; however, others have observed muscular dystrophy in some CC061 mice (due to a mutation in the dysferlin gene; http://csbio.unc.edu/CCstatus/index.py?run=availableLines). It should be noted that the CC016 strain has same dysferlin haplotype as CC061, but no gait problems. CC061 mice have also been found to have a split pubis, which could contribute to the waddling gait.

### Genetic diversity

Genes of the major histocompatibility region (known in mice as H2) have been associated with the persistence of TMEV infection^65^. Mice with some H2 haplotypes effectively clear the virus, while those with other H2 haplotypes develop demyelinating disease instead. Non-H2 genes are also known or suspected to contribute to TMEV susceptibility: for example, some congenic strains with TMEV-susceptible H2 haplotypes (e.g. the H2^s^ haplotype) on the background of a TMEV-resistant strain (e.g. C57BL/10) develop milder disease^66,67^. Two of these non-H2 genes have been identified as interferon gamma (*Ifng*) and interleukin 22 (*Il-22*)^68–71^. Both of these genes are located within ~20 kb of each other on mouse chromosome 10. Also on chromosome 10 is an enhancer-like long noncoding RNA (lncRNA) termed *NeST* (*nettoie Salmonella pas Theiler’s* [“cleanup Salmonella not Theiler’s”]). *NeST* has previously been found to contribute to TMEV susceptibility phenotypes by epigenetically regulating expression of *Ifng* ^72^. *NeST* RNA is encoded on the opposite DNA strand from *Ifng* and *Il-22*, including the entire length of *Ifng*.

Each CC strain is a mosaic of founder haplotypes. Each of the 5 CC strains used for this study possesses different H2 haplotypes, none of which have been previously defined as TMEV-susceptible or resistant since TMEV infection has not been studied in 4 of the founder strains from which the H2 regions of these strains were derived. Furthermore, these strains collectively have 2 haplotypes for the portion of chromosome 10 that harbors *Il-22, Ifng*, and *NeST*. Three of the 5 CC strains (CC013, CC019, and CC041) share the same haplotype for *Il-22*, *Ifng*, and *NeST*, inherited from the NZO/HILtJ founder strain, while CC016 and CC061 share a different haplotype for these loci. CC061 mice inherited this haplotype from the TMEV-resistant C57BL/6 J founder strain, which could have implications for the reduced severity of TMEV-induced disease experienced by CC061 mice. In CC016 mice, this haplotype is from the founder strain 128S1/SvImJ. These haplotypes consist of identical SNP genotypes and have 94% sequence identity; therefore it is likely CC016 mice also experienced a “buffering” of TMEV susceptibility, regardless of what risk their H2 haplotypes might have conferred. In fact, the *NeST* genotype has a powerful influence on whether a particular strain can clear the TMEV infection^72^, and the fact that CC016 and CC061 share the same *NeST* genotype strongly suggests that they were more resistant to TMEV than the other strains.

It is important to note here that strains have historically been classified as TMEV “resistant” or “susceptible” based on how quickly they can clear the virus. Those considered “resistant” clear the virus quickly; “susceptible” strains exhibit TMEV persistence leading to progressive demyelination. The level of viral RNA measured in CC061 mice during the acute phase resembled that measured for CC013 and CC019 mice (Fig. 2), but it is likely the CC061 mice were able to *clear* the virus more quickly than, for example, CC041 mice. This definition of resistance/susceptibility has been used throughout this paper to maintain consistency with over 30 years of TMEV literature; however, the diverse phenotypes presented by CC strains in this small preliminary study call for updated classifications of TMEV response. For example, some strains may be classified as “tolerant”, meaning infected mice could have a high viral burden but no clinical phenotypes. Future studies involving more CC strains are expected to reveal additional TMEV-induced phenotypes, which will enable expanded categorization of TMEV response types.

### Similarities to human diseases

i. *Amyotrophic lateral sclerosis*.
  Motor neuron destruction is a hallmark of ALS in humans^73,74^, and viral infection has long been considered a possible risk factor for ALS, as reviewed in detail in^6,11^. Indeed, Theiler’s virus RNA has been found in central nervous system tissue of ALS patients^25^. CC013 mice demonstrated forelimb weakness, which has also been observed in mouse models of ALS such as the “wobbler” mouse^75,76^. The forelimb weakness in this model of ALS is caused by the degeneration of motor neurons in the brainstem and the spinal cord. The permanent, profound hind limb paralysis demonstrated by CC041 mice following TMEV infection may also indicate substantial motor neuron damage. A longer observation period would be necessary to determine whether the TMEV-induced disease experienced by CC013 and CC041 mice reflects the etiology of ALS, as some mouse models of ALS do not show distinct ALS-like symptoms until 10–11 months of age^77–79^.
ii. *Epilepsy*.
  Epilepsy is a common neurological disorder, affecting approximately 1% of the population worldwide^80^. The condition can manifest in many ways across all ages^81^. Infections by several types of viruses are recognized as risk factors for epileptic seizures^7,8,13^, and genetic factors also contribute to this risk; for examples, see refs^82–84^.
  Seizures and seizure-like activity observed in 4 of the 5 strains studied did not present in the same ways. This suggests that CC mice could represent the variability seen in human epileptic conditions by modeling seizures with different etiologies. The fact that the seizures did not persist in the mice studied here does not preclude the possibility of other CC strains showing long-lasting, recurrent seizure activity: TMEV infection has been shown to cause spontaneous seizures several months post-infection in C57BL/6 mice^24,85^.
iii. *Multiple sclerosis*.
  Risk factors for MS include genetic background, especially MHC haplotype^86^ and environmental factors including viral infection^1,2,4,5,9,10,12,14^. The phenotypes resulting from TMEV infection of SJL mice parallel human MS in a number of ways, including chronic central nervous system inflammation involving CD4+ and CD8+ T cells, B cells, and macrophages^19,87^; sex-influenced disease phenotypes^60^; and clinical symptoms such as incontinence and gait disorders^67,88^. We observed gait changes suggestive of early symptoms of demyelination in 2 of the 5 CC strains studied: CC019 and CC041. Histopathology showed signs of axonal degeneration, typically seen with TMEV-induced demyelinating disease. However, spinal cord atrophy, which has been linked with severe disability in MS, occurs later during the chronic phase of TMEV infection^89^ but was not observed in any mice of this study.
iv. *Parkinson’s disease*.
  Parkinson’s Disease (PD), the second-most prevalent progressive neurodegenerative disorder in humans (behind Alzheimer’s disease), generally occurs later in life and is characterized by bradykinesia, postural instability, gait issues, and tremors^90^. Viral infection can influence the development of PD in humans e.g., refs.^91–98^. PD-like symptoms in mouse models resemble some of the signs seen in the TMEV-infected mice studied in this experiment, including reduced stride lengths and locomotion^79^. However, in many PD mouse models, typical Parkinsonian phenotypes such as tremors and rigidity are not observed until mice are considerably older – up to 18 months of age^77,99^. In the present study we evaluated Group A mouse phenotypes until approximately 5 months of age. Although we cannot conclusively declare that any of the 5 strains of mice we investigated presented with signs of PD, the phenotypic similarities we observed encourage more study.
v. *Other neurological conditions*.

Finally, there is the possibility that the TMEV-induced phenotypes presented by CC mice do not model any specific human condition. It is possible for a single virus to produce neurological phenotypes that resemble multiple conditions. For example, infection with the Coxsackie B virus has been found to cause a variety of defined neurological conditions including encephalitis and meningitis, but has also been known to cause nonspecific neurological sequelae^3^.

Furthermore, TMEV infection did not always produce signs of obvious neurological dysfunction. With regard to time spent on the rotarod and numbers of vertical plane entries (rearing), it is worth noting that TMEV-infected CC061 mice seemed to perform better – i.e., in a way more to be expected from healthy mice – than uninfected CC061 mice. Rotarod and rearing activities would be expected to show evidence of the hind limb weaknesses experienced by infected CC061 mice, but instead these mice appeared to exaggerate their physical capabilities when tested, almost as if to compensate for the effects of the infection. Perhaps the increased rearing could be due to lasting damage to the hippocampus during the acute phase of infection, which would have caused the mice to experience memory loss^54^; perhaps the mice reared up more to investigate their surroundings because they had trouble processing the unfamiliar environment or were experiencing anxiety. This behavior might parallel that which is sometimes associated with cognitive impairments that present in some human neurological conditions such as MS^100–102^. Of course, it is critical for rotarod and open field measurements to be interpreted in context with observational data and other quantitative analyses such as DigiGait.

## Conclusions

The CC is a powerful resource for studying diverse responses to pathological agents such as viruses. In this study, we found evidence that 5 CC strains are differently affected by TMEV infection, providing proof of concept to support the use of CC strains to study variation in TMEV-induced neuropathological changes. Future studies should allow more time for infected mice to develop clinical signs, as this may reveal more details about neurological sequelae. Also, cytokine and gene expression levels must be considered in the future to better understand how TMEV infection induces disease. Study of both sexes and both phases of TMEV infection is necessary to more completely characterize how genetic background affects the ultimate outcome of TMEV-induced diseases. The experiments described here underline a need for further investigation into how TMEV infection contributes to disease pathologies on different genetic backgrounds, particularly as such experiments may help us better understand similar conditions in humans.

## Materials and Methods

### Ethics statement

All animal care protocols were in accordance with NIH Guidelines for Care and Use of Laboratory Animals and were approved by the Texas A&M University Laboratory Animal Care and Use Committee (AUP 2014-0050).

### Mice

Two groups of mice were evaluated. Group A consisted of 35 mice representing 5 different CC strains, selected based on availability (CC013, CC016, CC019, CC041, and CC061; Table 1)^103–106^. Mice in Group A were monitored for 3 months (87–94 days post-infection; dpi) to evaluate long-term phenotypic differences.

**Table 1:** 72 TMEV-infected and control mice from 5 CC strains were used in these experiments.

Strain	Infected Female	Infected Male	Control Female	Control Male	Total
**CC013**	0 | 2	4 | 2	0 | 2	4 | 2	8** | 8**
**CC016**	2 | 0	0 | 0	0 | 0	0 | 0	**2 | 0**
**CC019**	3 | 3	4 | 3	1 | 2	1 | 2	**9** **| 10**
**CC041**	4 | 3	0 | 2	5 | 2	0 | 2	**9 | 9**
**CC061**	3 | 3	2 | 3	1 | 2	1 | 2	**7 | 10**
**Total**	**12 | 11**	**10 | 10**	**7 | 8**	**6 | 8**	**35 | 37**

Numbers of mice included in Group A are listed to the left of each cell; numbers in Group B are listed to the right of each cell.

Group B contained 37 mice, including 19 females and 18 males, from 4 of the 5 strains represented in Group A (CC013, CC019, CC041, and CC061). To identify phenotypic differences related to the acute phase of TMEV infection, roughly half of the Group B mice (2 females and 2 males per strain, including 1 infected of each sex) were evaluated for 7 dpi and the remaining mice (2–3 mice of each sex per strain, including 1–2 infected per sex) for 28 dpi.

### Infection

At 4 weeks of age, female (*n* = 23) and male (*n* = 20) mice were anesthetized by isoflurane inhalation (MWI, Meridian, ID) and injected intracerebrally with 5.0 × 10^4^ plaque-forming units (PFU) of the BeAn strain of TMEV (American Type Culture Collection [ATCC] VR 995, Manassas, VA) in 20 µl of PBS placed into the right mid-parietal cortex at a depth of approximately 1.5 mm^43,107^. Sham-infected mice (*n* = 15 females and 14 males) were anesthetized and injected with PBS only.

### Genotyping and haplotype reconstruction

Genotyping of CC strains has been described previously^108^. Genotype data is publicly available at the CC Status website (http://csbio.unc.edu/CCstatus/index.py?run=AvailableLines).

### Phenotyping

i. *Weight*
  Mice were weighed daily for at least 7 dpi, and at least once a week thereafter.
ii. *Seizures*
  All mice were monitored daily for neurological abnormalities including handling-induced behavioral seizures. Seizure activity was scored using the Racine scale, as follows: stage 1 – mouth and facial movement; stage 2 – head nodding; stage 3 – forelimb clonus; stage 4 – rearing with forelimb clonus; stage 5 – rearing and falling with forelimb clonus^109^.
iii. *Clinical scoring*
  Mice were assessed daily for clinical signs of neurological disease, using a scoring system known to correlate with histological scores^38^. Scoring was based on established markers for evaluating TMEV disease, including ruffled fur, hunched posture, grooming, and limb weakness. The severity of each of these markers was scored on a scale of 0 to 6, with 0 being normal and 6 being moribund^38,43^. Ruffling, or piloerection, is a clinical sign of encephalitis along with hunched posture, lethargy, and sunken eyes^38,43,110,111^. Hunching in mice gradually increases with disease progression^38,43,112^. Another typical sickness behavior in mice is changes in amount of grooming. Grooming was measured only for Group A mice, by recording videos of individual mice in open-field arenas for 10 minutes and measuring time spent performing grooming behaviors. We evaluated limb impairment by timing how long mice clung to an inverted metal grate and how well each limb grasped the grate as described in detail in^110,112^.
iv. *Gait assessment*
  Changes in stride have been previously correlated with neurological sequelae in rodents^113–115^, including disease progression during TMEV infection in mice^39^. Mice in Groups A and B were evaluated separately, using different methods and equipment as described below.
  Foot print - Group A: Footprint analysis provides an objective, quantitative evaluation of neurological impairment in rodents^39,113–116^. To identify changes in stride length, stride width, and overall gait, fore and rear paws of Group A mice were painted red and blue, respectively, with nontoxic paint as previously described^39,116^. Mice then walked along a strip of white paper inside a defined walkway approximately 7 cm wide by 90 cm long with walls 10 cm tall on each side. The resulting foot prints were scanned electronically and distances between prints were measured using ImageJ US National Institutes of Health, Bethesda, MD^117^. Stride lengths and widths for both forelimbs and hind limbs were measured for a minimum of 6 sequential strides, as described previously^39^. These assessments were performed weekly for 7 consecutive weeks starting at 7 weeks post-infection (p.i.).
  DigiGait - Group B: We used DigiGait (Mouse Specifics, Boston, MA, USA) for quantitative, noninvasive analyses of various gait parameters, as previously described^118,119^. DigiGait is especially useful for delineating subtle neurological phenotypes^118–121^. Measurements were taken at 27 dpi. After the mice underwent an initial training period, we recorded mice walking at a speed of 19 cm/s for a minimum of 6 consecutive strides. For all measurements we included age- and sex-matched control mice for reference. Measurements are presented as average values from both forelimbs and both hind limbs.
v. *Rotarod*
  Motor impairments were evaluated in Group A mice using rotarod^39^ at ~7 weeks p.i. Each mouse was placed on a rod (5 cm in diameter; Ugo Basile, model 7650, Varese, Italy). This rod rotated at 4 rpm initially, and every 30 s for a total of 300 s the speed of rotation was increased by 4 rpm. For each mouse, the latency and rod speed at the time the mouse fell were recorded. Each measurement was repeated three times, with a 15 min break between each trial. Average values across strains were used for comparisons; average values for separate sexes for each strain were also evaluated when available.
vi. *Open field*

The locomotive behaviors of Group A mice were evaluated by placing individual mice in a Plexiglas arena 43 cm long by 43 cm wide by 43 cm tall equipped with an activity monitoring system (Tru Scan; Coulbourn Instruments, Holliston, MA). Under ambient room light, mice were scored for aspects of horizontal and vertical movement for 30 min^38^.

### Euthanasia and tissue collection

Group A mice were euthanized 87–94 days post-infection, and Group B mice were euthanized either 7 or 28 days p.i. by intraperitoneal (i.p.) injection of a lethal dose of Beuthanasia special 150 mg/kg (Schering-Plough Animal Health) as described^111^. Mice were perfused through the left ventricle with phosphate-buffered saline (PBS) followed by 10% formalin in phosphate buffer at pH 7.2. A systematic necropsy was performed. For each mouse, one cerebral hemisphere was snap-frozen on dry ice and then stored at −80 °C for RNA extraction. The spinal cord and the contralateral cerebral hemisphere were fixed overnight in 4% paraformaldehyde for histologic examination.

### RNA extraction

Total RNA was extracted from brains and archived at −80 °C for each mouse, using Maxwell® 16 automated equipment for nucleic acid purification with LEV simplyRNA tissue kit (Promega, Sunnyvale, CA). Quantification and quality checking were performed using a Cytation 5 Imaging Multi-Mode Reader (BioTek).

### TMEV quantification

Total RNA (500 ng) was reverse transcribed to cDNA using SuperScript II (Life Technologies, Grand Island, NY) in 8 µl reactions; after RT, cDNA was diluted 1:5 in nuclease-free water. All qPCR reactions were performed in duplicate using SYBR Green. Each 20 µl reaction contained 10 µl Power SYBR® Green PCR Master Mix (Life Technologies), 0.6 µl each forward and reverse primers (10 µM), 6.8 µl ultra-pure water, and 2.0 µl cDNA. Primers were designed using Primer3 software (http://biotools.umassmed.edu/bioapps/primer3_www.cgi ^122^). Primers for the housekeeping gene phosphoglycerate kinase (*Pgk1*) were generated using the sequence NM_008828.3: forward 5′-catggtgggtgtgaatctg-3′; reverse 5′-caaagccttggcaaagtagt-3′. TMEV primers were designed from the sequence of the DA strain of TMEV (JX443418.1): forward 5′-ggactctcgttgttgctgtg-3′; reverse 5′-tggtgtcagggttagtggag-3′. Relative TMEV expression data were calculated by the ΔΔCt method of Livak and Schmittgen^123^, normalized to *Pgk1*. TMEV RNA levels were measured only for Group B mice, to evaluate viral clearance at 7 and 28 dpi (representing acute and chronic stages of infection).

### Histological evaluation of central nervous system

For Group B mice, brains and spinal cords were sectioned and processed for staining. Briefly, level 2 (frontoparietal cortex and caudate-putamen), level 3 (frontoparietal cortex, hippocampus and thalamus), level 4 (occipital cortex and midbrain) and level 6 (cerebellar peduncles and pons) of the brain, and cervical, thoracic and lumbar sections of the spinal cord were sectioned as previously described in^124^. H&E-stained slides were evaluated histologically without prior knowledge of experimental treatment. Slides were viewed using an Olympus BX43-F microscope at 40x magnification with a DP73 camera, ND filters and CellSens Standard software.

### Statistics

Quantitative data (including DigiGait, open field and clinical score data) were analyzed using factorial ANOVA (JMP version 12)^125^ of least squared means with sex and infection status and their interactions as variables. When appropriate, post hoc comparisons were performed using multiple comparison testing (Tukey method) and Fisher’s least significant difference (LSD) test.

In all cases, *p* ≤ 0.05 was considered significant.

### Data availability

The datasets generated during and/or analyzed during the current study are available from the corresponding author on reasonable request.

## Electronic supplementary material

Supplementary Dataset 1

Supplementary Dataset 2

Supplementary Dataset 3

Supplementary Dataset 4
